# Origins and localization of Tai Lue food culture in Northern Thailand

**DOI:** 10.1186/s42779-023-00179-2

**Published:** 2023-06-01

**Authors:** Sansanee Krajangchom

**Affiliations:** Center of Tourism Research and Development, Multidisciplinary Research Institute, Chiang Mai University, 239 Huay Kaew Road, Suthep Sub-District, Mueang District, Chiang Mai, 50200 Thailand

**Keywords:** Culinary culture, Ethnic food identity, Localism, Northern Thailand, Tai Lue

## Abstract

This qualitative investigation explores the cuisine of the Tai Lue people, an ethnic minority group in Northern Thailand. Through documentary analysis, field study and participatory action research, four traditional Tai Lue dishes were transformed for presentation to visitors. Community members developed these products in cooperation with a trained chef. The final products were marketed through a university podcast project and at a cultural fair. The results of the investigation were disrupted by the Covid pandemic, but the initial impact of the project indicates a prospective place in the wider post-pandemic environment for the indigenous cuisine of the Tai Lue. The following paper discusses this potential through the lens of the foodscape, regional development and authenticity.

## Introduction

A well-defined ethnic food culture helps ensure continued existence for minority indigenous groups in an increasingly homogenized modern society. From Catholic Goan women in Canada to the ancestral community of La Playa Renaciente in Cali, Colombia, and the mixture of ethnic groups in multicultural Singapore, minority communities around the world use their cuisine as a means of continuing traditional sociocultural practices in their everyday lives [1–3]. Food is especially ‘central to the ways migrants identify themselves both individually and collectively,’ providing familiarity and individuality [4] (p.25)]. It is also increasingly a means for economic survival and relevance of ethnic groups in capitalist societies [5–7].

Prior to the hiatus in global travel caused by the COVID-19 pandemic, food tourism was playing an increasingly important role in enhancing cultural identity and furthering local and national economic development. Bundit Anekpoonsuk found that more than a third of all tourism expenditure in Thailand was spent on food before global travel restrictions took their toll on the Thai economy [8]. Local vendors across the country were tapping into the marketing potential of traditional Thai gastronomy, which had been extensively promoted by the national government as a kind of culinary brand identity [9, 10]. However, when international tourists stopped coming, producers needed to focus on the other demographic in their target market: nostalgia-motivated domestic visitors [11].

The pursuit of local uniqueness remains popular among tourists in Thailand and will provide the re-entry point for many businesses as they return to a post-Covid normality [12]. When traveling, domestic tourists look for identity through food [13]. Thailand is rich in plants with positive nutritional and medicinal properties. Various localities have benefited from this and have become synonymous with their food tourism programs [14, 15]. Health and well-being have also become popularly associated with this phenomenon and many destinations now focus on the identity of their cuisine as a tool for economic development [16]. The added focus on domestic cuisine could also provide increased opportunities for historically marginalized ethnic communities, whose food culture is an integral part of local identity. Food provides an experience of otherness that “is understood within the Asian context through ethnic diversity and social changes in the region” [17] (p.3)]. By using food as a medium for storytelling, minority ethnic groups can simultaneously strengthen their own identity and reap the economic benefits of a greater presence within the Thai tourism sector.

The Tai Lue ethnic group is one minority community with a unique culinary culture and set of traditions who could benefit from this scenario. The Tai Lue people have adapted to life as a component of Thai society, yet have retained their distinctive identity. They live in the upper northern part of Thailand and are descendants of migrants from Xishuangbanna, currently an autonomous prefecture in China's southwestern Yunnan province, bordering Myanmar and Laos. The spoken language, dress, belief system, performing arts and agricultural practices are all consciously preserved, but their cuisine holds particular potential for attracting domestic tourists [18, 19].

Tai Lue cuisine is predominantly vegetarian, which complements the modern drive toward more sustainable consumption patterns. Vegetables are planted in kitchen gardens surrounding the typical Tai Lue house. Tai Lue people usually cook simple dishes using the locally-sourced natural ingredients grown in these kitchen gardens or foraged from nearby forests [20]. However, their culture and cuisine are relatively little known. The project this paper discusses was therefore designed to raise awareness of local Tai Lue food and add value to the local economy through the development of distinctive dishes and promotion of a well-defined Tai Lue eating culture among wider Thai society. This paper is therefore one of few scholarly attempts to introduce Tai Lue cuisine to broader academic audiences. This was facilitated by the Chiang Mai University Podcast Project to benefit those interested in developing local indigenous cuisine and provide a new culinary perspective for tourists. It is hoped that the project will lead to further study of the Tai Lue eating culture and open opportunities for the Tai Lue people of Northern Thailand to participate in the promotion of local traditional culinary knowledge, cultural capital and tourism.

## Literature review

Food has long been recognized as an essential component of national culture and identity. According to Smith, it creates a sense of authenticity and is a symbol of ethnicity [21]. Food is therefore promoted by governments around the world as a form of political capital that creates jobs and income [22]. This strategy has been adopted by Thailand, whose successful attempts at ‘gastrodiplomacy’ have led to the globalization of Thai food and (pre-Covid) to a dramatic increase in food tourism to the country [23].

Food tourism began to emerge in the late twentieth-century as tourists grew to value cultural experiences over packaged holidays [24]. In academic literature, it received treatment by a number of different disciplines, but the common theme was the link between food and culture [25]. Food often contributes toward a romanticized image of the culture to be found in tourist destinations [26]. This is due to the perceived local authenticity it provides and a rebellion against globalization [27]. Its recent growth is part of a general increase in special interest tourism, itself either a reaction to the perceived homogeneity caused by an ever-more interconnected world [26, 28] or a byproduct of higher standards of living [29–31].

Increasingly, studies are recommending a shift of emphasis from the traditional perspective of food as a component of tourism into food as the primary attraction. The consequence is the development of more unique culinary experiences and stronger stakeholder involvement in food identity development [32]. This fits into the notion of a ‘foodscape’, a food culture “shaped, influenced, transformed by social practices…by political and legal institutions, by economic decisions, and by relations of power within food systems” [33] (p.16)]. Food is therefore reimagined as the fulcrum of a dynamic sociocultural construct tying together place, space, people and meanings—it is being “re-territorialized” [34, 35] (p.1466)]. Within these confines, food culture is a non-static, evolving part of society that merits simultaneous conservation and development alongside other aspects of tourism [36, 37].

In a recently published analysis of the foodscape and co-creation activities, Park and Widyanta found that tourists are beginning to be recognized among researchers for their contribution to the evolution of the foodscape in tourist destinations [37]. Cooking classes and wine tasting have taken the primary early focus in this growing body of research, but the scholarship is in its infancy [38–40]. However, because of their focus on co-creation, most studies tend to be consumer-centric in their analysis of food tourism [37, 41–43]. Comparatively few studies consider the role of the local producer in the foodscape evolution; fewer still consider the role of ethnic minority communities.

This is a significant area for regional development, which has primarily been linked to food through top-down initiatives such as the European Union’s Protected Designation of Origin (PDO) and the Protected Geographical Indication since the early 1990s (PGI) [44]. National governments have also tapped into food tourism “as a scalable cost-effective means of local and regional development, with the potential to strengthen identity, enhance appreciation of the environment and encourage the regeneration of local heritage and the local economy” [22] (p.96)]. However, in the last decade, there has been a greater focus on the role of local actors in sociocultural conservation and the collective dimension of regional development through food [45–48]. So-called terroir restaurants have emerged as spaces for the conservation of traditional cuisine, while slow-food movements and government initiatives champion local produce [31, 49, 50]. The link between food and territory provides an economic opportunity to improve individual enterprises but also carries the potential for entire communities and regions to benefit from tourism riches. Nonetheless, in many areas, a lack of centralization, lack of awareness and lack of trust among stakeholders prevent the success of regional development through food [51, 52]. There is also danger in falsification of traditional practices through catering to tourist demand rather than organically developing existing cultural practices. Some early innovations in food tourism have led to the development of traditional cuisines and the reengineering of traditional food production spaces for tourist consumption [25]. This has contributed to the commodification of intangible heritage, dilution of traditional culture and reduction in authenticity [53]. These authors are conscious that the present investigation threatened to do the same and, in order to try to mitigate this risk, fully involved members of the local Tai Lue community in all stages of the research and development process.

## Methodology

Tai Lue people live in several communities in Northern Thailand. For this investigation, two of these communities were purposively selected as the research area: Ban Luang Nuea, Luang Nuea Sub-District, Doi Saket District, Chiang Mai Province and Ban Phae Ton Yang Ngam, Ban Thi Sub-District, Ban Thi District, Lamphun Province. The residents of these communities were considered the population for this investigation. Through purposive sampling, a research sample of local residents, chefs and tourism administrators was selected from the research area. These individuals recalled oral histories and shared opinions on Tai Lue cuisine through informal and formal interviews. The researcher also gathered information from documentary review and observation in the target communities. This is therefore a documentative and descriptive work predominately aimed at working with Tai Lue people to uncover and develop their traditional food in four stages.

In Stage 1, initial research data on the local Tai Lue food culture were collected from informants through field visits. ‘Ideation Sessions’ were held and interviews were hosted with village elders, Tai Lue community members, Tai Lue restaurant owners, Tai Lue chefs and local tourism entrepreneurs in each province. The purpose of these discussions was to learn about existing Tai Lue cuisine, gather local opinions on ways to develop the food culture and seek approval for any potential food innovations. Content analysis was conducted on the Tai Lue ethnic eating culture and traditional knowledge to find cultural artifacts and provide information for creating stories linking local food and the food culture of the Tai Lue ethnic group of the northern region.

In Stage 2, through observation in the communities and discussions with local chefs, the researcher explored the raw materials available in the area and considered their potential for adding value to the cuisine in accordance with the needs of the community. Tai Lue people were then consulted in the development of dishes with expert chefs. The food composition was also analyzed (proximate composition and energy—protein, fat, carbohydrate content) in consideration of modern health trends, sustainability and marketing potential, although this information has been omitted from the paper in the interests of brevity and focus—a future paper will discuss the comparative nutritional benefits of indigenous Tai Lue cuisine. A menu for Local Tai Lue cuisine was prepared for marketing through the Chiang Mai University Podcast Project.

In Stage 3, the researcher developed story-telling scripts that combined the Tai Lue culture and cuisine using information collected from key stakeholders during field visits, ideation sessions and interviews. Following approval from Tai Lue representatives, the content of local food and culture of the Tai Lue ethnic group was subsequently presented through the Chiang Mai University Podcast Project by the researcher and selected community representatives.

In Stage 4, the researcher and local Tai Lue people presented the findings of the investigation at a Northern Thai festival celebrating local culture. The eating culture and food wisdom of the Tai Lue ethnic group was placed in the context of Northern Thai cuisine and linked to local food products. The Local Tai Lue Cuisine menu was cooked and presented by a specialist Tai Lue chef. Throughout the research process, data were categorized typologically and validated by methodological and source triangulation. The approved results were analyzed inductively and are presented below as a descriptive analysis.

## Results

Academic consensus suggests that the Tai Lue migrated from Xishuangbanna Dai Prefecture in Southern China, formerly known as Chiang Hung [54–56]. This was supported by evidence found in the two Northern communities. “Pap Kham Kaew” is a Tai Lue lyric book of unknown origin that tells the creation story of Prathom Kalp Brahma. According to the local book (of which copies were found in both Chiang Mai and Lamphun), Lue people or Tai Lue lived in the Lue Luang (in Chinese: Lue Chaeng) area. There is no evidence of where this settlement was located. Later the people escaped an epidemic in Lue Luang, moving south to Nong Sae, modern-day Kunming. Research respondents unanimously believed that Kham Daeng, a female leader, brought the first settlers and hundreds and thousands of people continued to migrate south over time. According to legend, it took 2 years for the original settlers to cross rivers, mountains and plains until they reached the Mekong River Basin. Once there, they established a new settlement called Lue City. At present, the Tai Lue people are generally scattered in lowlands and valleys in the Mekong River Basin and were the first Tai people to migrate from southern China. The local people are extremely proud of these origins and were keen that any story-telling initiative reflected and celebrated their ancestry.

### Food of the Tai Lue

The Tai Lue ethnic group has a simple eating style. The everyday diet is dependent on the naturally available resources in each season. The Tai Lue people mainly eat sticky rice as the staple product; however, the glutinous rice that the Tai Lue people eat is dark red in color (often called *Khao Kam* by other groups in Northern Thailand, from the family *Oryza sativa* L.). Most meals include some form of chili paste and steamed vegetables or vegetable curry. Common vegetables include cha-om (*Acacia pennata *(L.)* Willd*) and bok choy (*Brassica rapa* subsp. *chinensis*). There are three primary categories of dishes at the Tai Lue table: rice-based dishes, meat-based dishes and vegetable-based dishes.

While the Tai Lue use a variety of cooking methods, these are very similar to those used in Northern Thai cuisine, differing only in the name. There are eleven common methods: currying (locally called *kae*), roasting, boiling, deep-frying, steaming, grilling, stir-frying, salad, sautéing, baking and mixing into salads.

### Food according to festivals and traditions

As with many Asian cultures, food and calendrical festivals are intrinsically linked in Tai Lue communities. Tai Lue people cook food to make merit and entertain guests. The dishes created for this purpose are mostly those eaten in everyday life. Many ritual foods are rice-based sweets or desserts, including *khao dok so*, *khao tan*, *khao pong* and *khao tom kob*. One important traditional food is *khanom pad*, a kind of caramel dish made from a combination of rice flour, coconut milk and sugar. The ingredients are brought to a simmer until sticky and wrapped in banana leaf. Another type of dessert that the Tai Lue people often make during festivals is *khanom jok*, a filled sweet made from glutinous rice flour kneaded with sugar and stuffed with a grated coconut and sugarcane juice mixture. This is often offered to monks alongside the savory food prepared for merit-making.

In addition to the dishes consumed at ritual events, the Tai Lue people practice animist beliefs. Their tradition of paying respect to ancestral spirits also includes the presentation of sacrificial foods, including boiled chicken, boiled eggs and a boiled pig’s head. These offerings are accompanied by liquor, flowers and incense sticks. A bamboo stick tied with a red cloth is placed to mark the location of the sacrificial ceremony (called a *talaew*) and food clearly plays a hugely important role in the Tai Lue psyche.

### Eating culture of the Tai Lue

**Fig. 1:**
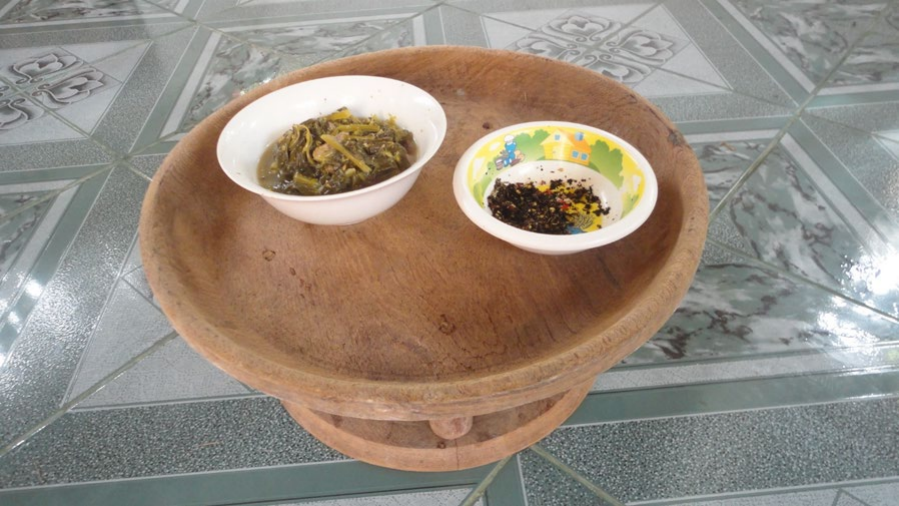
Traditional Tai Lue *khantoke* made from teak

At *Tai Lue* mealtimes, the food is contained in dishes and placed on the *khantoke*, a circular platter. The housewives (who have generally done the cooking) lay out mats for the diners and place the finished *khantoke* in the center. The rice is not usually stored on the platter and is instead brought in containers separately to the main dishes. Some families also bring water bowls for guests to wash their hands and hand towels for cleaning. When eating, it is traditional to allow seniors to eat first, which is an expression of respect. This is a similar ritual to traditional Thai households, although it should be noted that the material of traditional Thai *khantoke* and rice containers is bamboo. Due to the *Tai Lue* proximity to the forest, their *khantoke* and rice vessels are made of teak and coconut shell (Fig. 1).

The traditional *Tai Lue khantoke* are created using a lathe. The carpenter takes a large piece of teak, cuts it accordingly and turns or chips it into shape. Lacquer is then applied for durability. The object is sometimes called a *satok* depending on the local dialect. There are three sizes of *khantoke*: *Khantoke Luang* (large—from 25 to 50 inches in diameter) is popularly used in the courts, palaces of the northern lords and temples. The size of these *khantoke* facilitates hosting guests in large numbers. Locals use them to bring rice, fish and vegetables as offerings to make merit at the temple. *Khantok ham,* or *khantoke tharam*, is a medium-sized khantoke about 17–24 inches. The word *ham* or *tharam* is an ancient Thai word that appears in the inscriptions of King Ramkhamhaeng. This size of *khantoke* is often used in large families or for deputy monks. The *khantok noi* is 10–15 inches wide and used for small kitchens. These are easy to find in Northern Thailand, and relatively inexpensive. They are also sold in combination with food coverings. One advantage of teak as the material is its resistance to insects and dust. Rice containers are commonly made from woven bamboo and come in sizes of 10 inches tall (small), 20 inches tall (medium) and 30–40 inches tall (large) with a diameter of about 10 inches. They are popularly made with a woven lid to prevent flies or dust. There is a hole in the container for a thread to be inserted into the cap so that the container can be carried. The *khantoke* eating style of the *Tai Lue* usually requires the male diners to be seated cross-legged on the floor and the female diners to be seated with bent knees and the feet pointing away from the center of the group.

### Transforming Tai Lue Cuisine

Local villagers were keen to add value to their local food so that it would be more acceptable to visiting tourists. “We have a rich and unique cuisine, but it does not always seem appealing to newcomers” (Interviewee 1—Tai Lue village elder, personal communication, 2022). Some of the younger members of the community were also conscious that the presentation of the food “is not appealing to modern visitors, who are accustomed to eating in stylish restaurants” (Interviewee 2—Tai Lue resident, personal communication, 2022). As one young person remarked, “the taste is delicious, but the dish is not appetizing” (Interviewee 3—Tai Lue resident, personal communication, 2022). In order to create more value for local food to allow Tai Lue chefs to compete for tourist business, local producers were interested in developing fusion cuisine that retained the original Tai Lue identity but was also presented in different ways. It was believed that this value creation could become a marketing tool that would lead to further food product development, distribution, advertising and marketing promotion and the ability to set higher prices. In response to this desire, Mr. Panupon Bulsuwan, the head chef of Black Kitchen Suchan was invited to the community and work with local cooks to develop a food menu of Local Tai Lue dishes. After consultation with Tai Lue locals, four items were chosen: *Mee Toon Kob Son Rub*, *Sa Pli Spring Rolls*, *Boneless Snakehead Fish Stuffed with Pork* and *Nam Prik Kaeng Khae*. Details of each dish are described below.

**Fig. 2:**
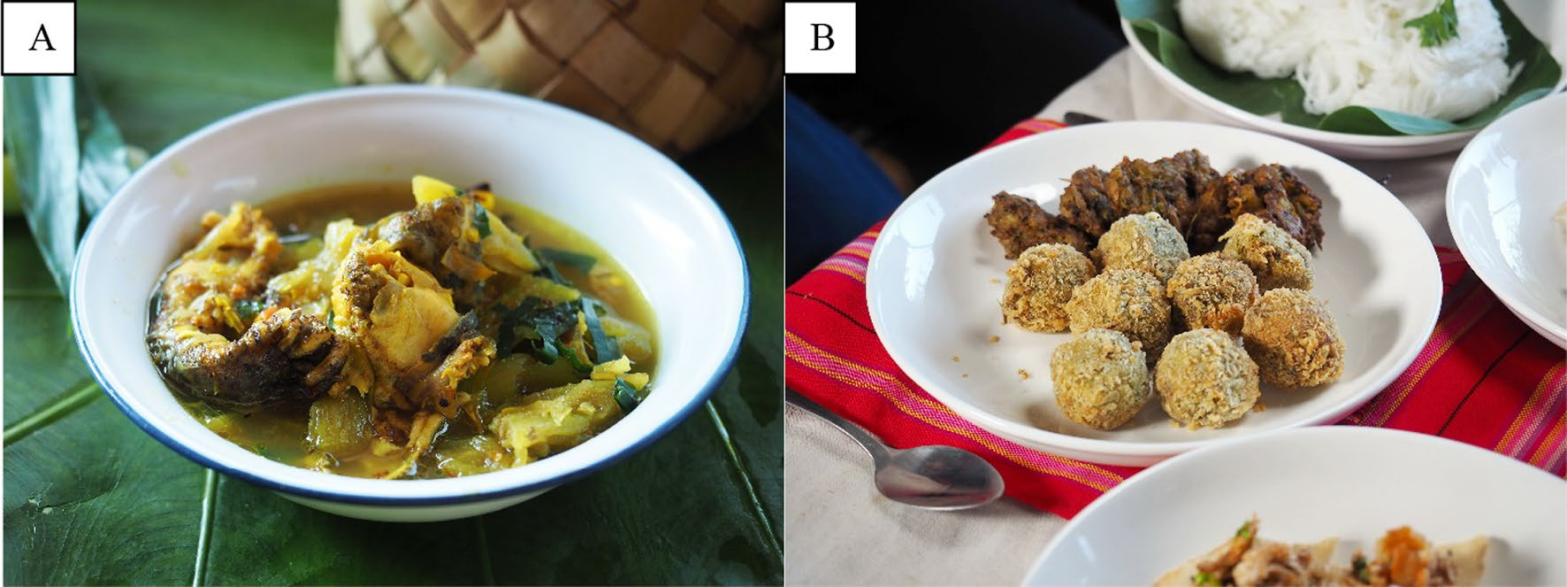
**A** Mee Kop Sai Toon and **B** Mee Toon Kop Son Rub

*Mee Toon Kop Son Rub* is an extension of *Mee Kop Sai Toon*, which is considered a winter menu from the rice fields of the Tai Lue community in Ban Luang Nuea (Fig. 2). The ancestral lifestyle of the local people is closely tied to farming, and frogs are commonly found in the fields, especially during winter when the rice is ready to harvest. At this time, frogs are easier to catch and sweeter than in other seasons. Therefore, people like to incorporate them into a variety of dishes, including *Mee Kop Sai Toon,* a curry made from the stem of a locally found plant, *toon* (*Colocasia gigantean Hook.f*). In this transformation of *Mee Kop Sai Toon,* the original ingredients (frogs, toon, young chilies, garlic, shallots, lemongrass, turmeric, ginger leaves, shrimp paste, fish sauce and vegetable oil) have been retained. The new recipe calls for the liquid to evaporate and the dry mixture to be coated in flour and deep-fried. The difference prior to cooking is that the frog meat is torn into smaller pieces and the bones are removed. After simmering down, the mixture is formed into balls, deep-fried until crispy and served with a dipping sauce made from fresh *toon*. The villagers created the name of the new dish. The flavor remains just as intense as the original curry, but it looks more appetizing for the unaccustomed palette and is “easier to eat on the go” (Interviewee 4—tourism entrepreneur, personal communication, 2022). Furthermore, the final dish is “more suited to visitors not used to eating frogs” (Interviewee 5—tourism entrepreneur, personal communication, 2022).

**Fig. 3:**
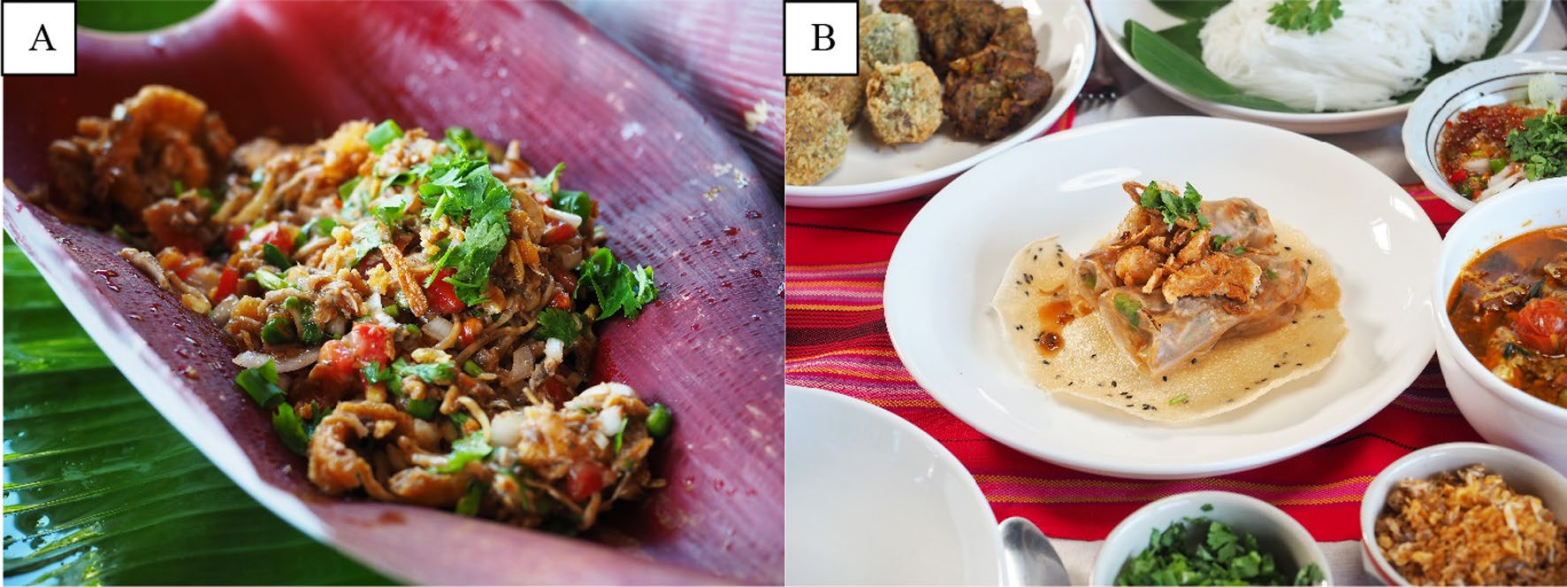
**A** Sa Pli and **B** Sa Pli Spring Rolls

*Sa Pli Spring Rolls* are a transformation of the traditional home-cooked Tai Lue dish, *Sa Pli* (Fig. 3)*.* The dish has a refreshing mixture of spicy and sour flavors and can be eaten in every season. However, during the rainy season the main ingredient, banana flower, will be particularly sweet, oily and crunchy, making it an ideal component for this spicy salad. The spring rolls still use the same ingredients, banana flower, red peppers, shallots, garlic, tomatoes and lime, but the fish type is interchangeable according to the preference of the chef. The soft parts of the banana flower are shredded and soaked in water. They are then mixed with lime juice to maintain freshness and color. Red chili, shallot, garlic and tomatoes are cut into small pieces and mixed with fish (typically mackerel), before adding the shredded banana flower in the last step. By wrapping this mixture in spring roll pastry, an additional layer of texture is added and, as with *Mee Toon Kop Son Rub,* the dish is “perfect for a snack on the road” (Interviewee 6—local Tai Lue chef, personal communication, 2022).

**Fig. 4:**
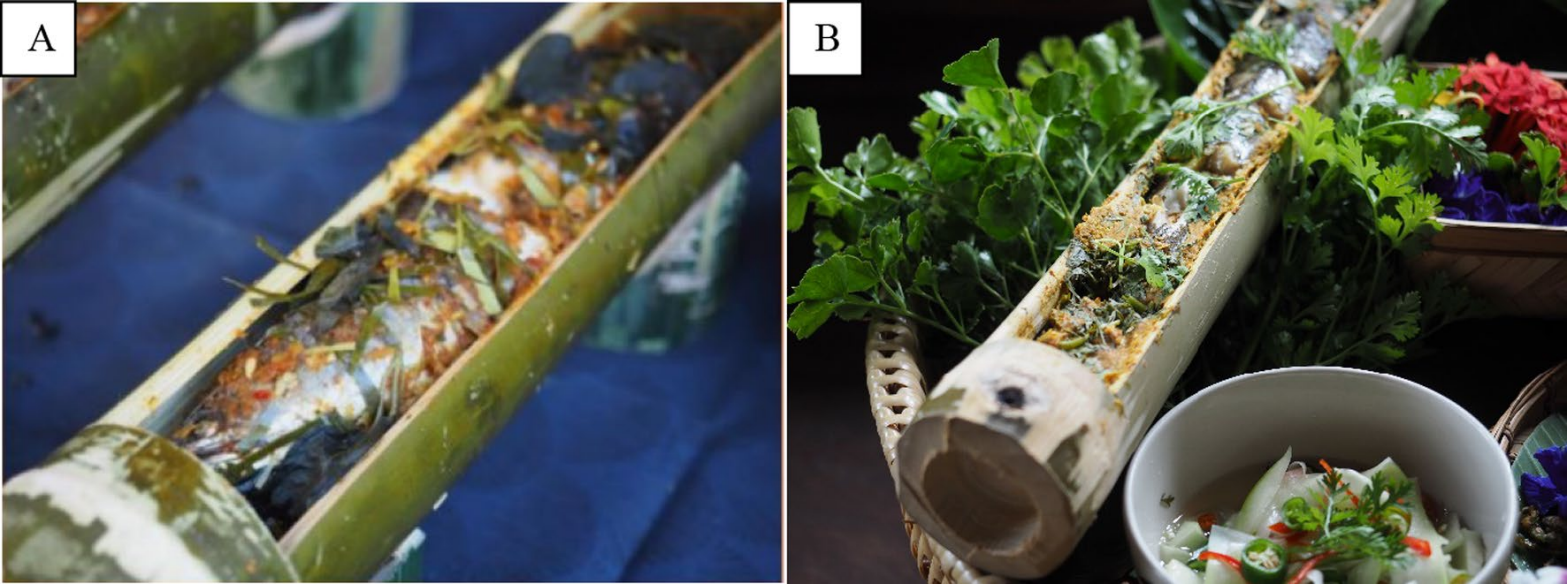
**A** Snakehead Fish in a Bamboo Case and **B** Boneless Snakehead Fish Stuffed with Pork

Boneless snakehead fish stuffed with pork is a transformation of *Lam Pla*, snakehead fish baked in a bamboo case in the Tai Lue style (Fig. 4). It is a delicious local food of the Tai Lue community in Ban Phae Ton Yang Ngam, and is locally classified as a ‘secret dish’ because it is often prepared, cooked and eaten by foragers searching for ingredients in the wilderness without common cooking utensils. Snakehead fish is the base ingredient and is combined with chili, garlic, shallots, turmeric, fresh coriander seeds, lemongrass, basil, Chinese cabbage and pandan leaves. The mixture is stuffed into a bamboo stem and baked, giving the dish a distinctive aroma from the bamboo. It has become a special dish that the villagers of Phae Ton Yang Ngam are proud to present when welcoming guests. Consequently, not a great deal has been transformed in the new dish so that the original identity could be preserved, although the bones of the fish are removed prior to cooking to facilitate easier consumption. Pork has also been added to the mixture to make it slightly more similar to a Thai dish named *Ho Mok Ping*. The resultant taste is “smooth and well-combined” (Interviewee 7—Tai Lue restaurant owner, personal communication, 2022).

**Fig. 5:**
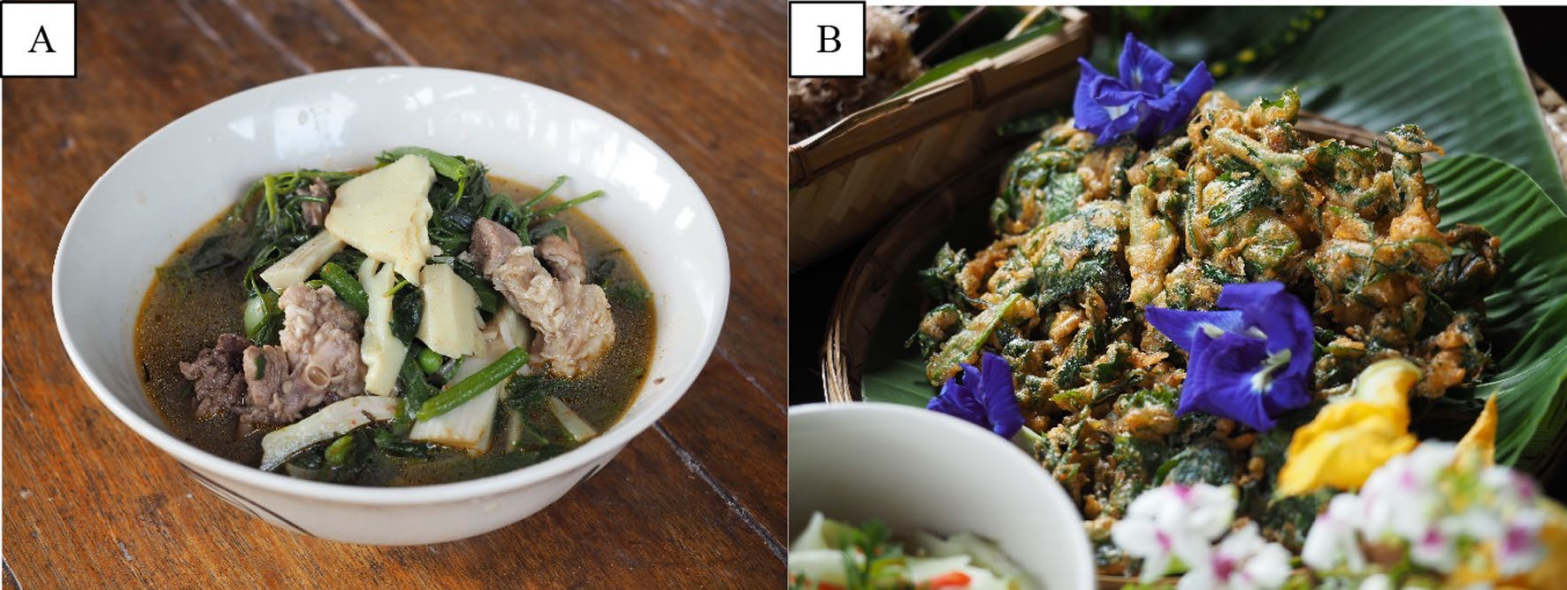
**A** Kaeng Khae and **B** Fried Vegetables with Nam Prik Kaeng Khae

*Kaeng Khae* is a curry found in local Tai Lue communities that contains 13 types of vegetable, including *pak wan* (*Melientha suavis*), rat-tailed radish, pumpkin shoots, gourd shoots, wind mushrooms (*Panus polychrous*), yard long beans, eggplants, parsley, vegetables, *pak khae* (*Sesbania grandiflora *(L.)* Pers*), *cha-om* (*Acacia pennata *(L.) *Willd*), *pak gan* (*Colubrina asiatica *(L.)* Brongn*), *pak siaw* (*Bauhinia Purpurea *Linn.) and other rare forest plants (Fig. 5). The traditional recipe uses banana flower, which has a bitter and sweet taste and helps to decorate the dish, making it more appetizing. The transformation is a partially deconstructed curry combining crispy fried vegetables with the curry sauce as a topping. This serves to change the presentation style, but importantly allows the diner to adjust the degree of spiciness to their preference. The nature of the dish made it a favorite among local children when they tried it and ensured that it can be eaten easily.

### Initial impact: the Chiang Mai University podcast and cultural fair

The dishes were introduced to the general Thai public through the Chiang Mai University Podcast project. Tai Lue community members connected people to their food and eating culture using stories of Tai Lue history in the north. This dissemination sought to simultaneously benefit social understanding of the Tai Lue ethnic group and provide economic opportunities for the local community. It was intended that increased publicity would generate income for people in the Tai Lue ethnic community from tourists interested in new Tai Lue recipes. It was also hoped that economic, social and environmental values could be created through the application and innovation of knowledge in Tai Lue food.

The Tai Lue people have applied the experiences from the project to welcome visitors to the community, including preparing and demonstrating “Dang-ngo Tai Lue” (a modification of the Tai Lue cuisine through a project between chefs and traditional knowledge teachers) for a research team visiting the area to survey and collect data on the Tai Lue Banthi fabric group and build community entrepreneurs. More tourists and interested people have also come to eat at the local restaurant, Krua Tai Lue. In the restaurant, there have been adjustments and additions to the menu in order to meet the consumer demands, such as re-sizing *khao jee* to be smaller and changing the menu options from frog to chicken for those who do not eat frogs. Information about activities that add value to food is shared through various web pages of the Tai Lue communities in Ban Phae and Ban Luang Nuea. Due to the COVID-19 outbreak, the “Creative Lanna Festival” was not held but the researcher consequently published the results of their research to the public at the Summer Book Fest 2021. It is hoped that a proper taste test can be conducted at the proposed 2022 Creative Lanna Festival and that consumer opinions of the transformed Tai Lue dishes can be gathered.

## Discussion

Throughout this investigation, interviews and observations constantly revealed the value of food to the Tai Lue people. Local people cherish their cuisine and are extremely proud of its identity. It provides a connection to the ancestral way of life and is intrinsically linked to a number of social and spiritual rituals. Actually, food is one way in which ethnic communities were able to retain a sense of traditions in the face of intense, systematic acculturation carried out by the Thai government in the early twentieth century [57]. In an explicit policy of nation building, “successive Thai governments adopted strategic policies of cultural hegemony to foster a singular national identity” [58, 59] (p.2)]. This process of Thaification led to the integration of ethnic groups into the majority *Tai* mainstream and caused the dilution of many traditional practices and ceremonies [60]. While traditional dress and arts have undeniably taken on a ceremonial role as younger people become more attached to modern Thai culture, the traditional cuisine has retained its originality. Dishes such as *Mee Kop Sai Toon* and *Sa Pli* continue to demonstrate the uniqueness of the Tai Lue and the everyday consumption shows that Tai Lue culture is alive.

The intention of this food transformation and marketing project was to “facilitate growing consumptive demands and increase profits,” as well as seek a more prominent place for the Tai Lue in a new, post-Covid tourism environment [53] (p.551)]. The early signs from Krua Tai Lue suggest this objective has been achieved, at least in the short term. However, there is clearly danger in external researchers arriving to reengineer food production spaces. Transformation of traditional kitchens into “places of tourist consumption…can provide more economic benefits for private stakeholders, but at the same time may provoke a commodification of immaterial heritage” [31] (p.45)]. Initiatives such as this one, while harboring good intentions, have been accused of diluting authenticity and destroying the culture they set out to preserve in the same way as those early Thaification measures [61]. One way in which this investigation differs from those criticized is the voice of local Tai Lue people. Throughout the process, villagers were involved in determining the direction of the research and culinary adaptations, which is essential for equitable research among minority communities [62]. There must also be consideration of what it means to be authentic. Culture is always in flux. It is unrealistic to treat any living social culture as a closed system, especially regarding cuisine. This was most expertly put by Lu and Fine [63] (pp.538–539)], who reflected that

From generation to generation, some culinary preparations and foodways absorb features of "alien" foods - perhaps a function of biological succession of foodstuffs, migration, technological change, shortages, or alterations in food-related ideologies (e.g., increased negative attitudes toward red meats, sugar, fat, or animal products; increased positive attitudes toward fish, turkey, or leafy greens). The vitality of a culinary system depends on its adaptability and flexibility. The maintenance of a food pattern does not depend on whether it is identical with an original model but on whether the "fundamental" characteristics of the food are defined as being continuously present, connected to core cultural beliefs, and recognized as a differentiated food pattern.

It is true that the fusion dishes created during this investigation are forced by a desire to appeal to perceived non-Tai Lue preferences, but there are two mitigating factors that should be considered: (1) the dishes were created by Tai Lue people—they are the Tai Lue interpretation of fusion cuisine and must continue to count as Tai Lue cultural products; (2) the traditional dishes continue to be eaten in everyday Tai Lue households. The fusion dishes are produced by restaurants for the mainstream Thai palette, giving them an insight into Tai Lue cuisine that they would not otherwise enjoy. Furthermore, the positive sentiments surrounding fusion cuisine are shared by similar studies in other regions of Southeast Asia: French-Lao fusion food is viewed as an empowering and conscious evolution of Luang Prabang cuisine [64]; in the Central Thai Plains, local vendors reimagine traditional cuisine for the tourist palate on a continuum of authenticity that is no less valid for its novelty [27]; Macanese fusion food is billed as part of the evolution of culinary identity on Macao [65]. This researcher is convinced that the traditional dishes will continue to be eaten in Tai Lue households because, as Montanari and Staniscia [35] concluded, culinary innovations are demanded in urban areas rather than the everyday rural environments in which the Tai Lue people live. In Tai Lue communities, authenticity and freshness of the ingredients remain the most important characteristics of food.

This concept is enhanced at a time of increased resistance to external change. Consumers are attracted by local peculiarities because they provide a unique experience detached from the increasingly homogenized society in which many live [66]. Through local innovations, the Tai Lue can appeal to and attract domestic visitors interested in a localized Thai experience that cannot be found elsewhere. This attachment to the particular geographic location should be emphasized and celebrated in much the same way as the French concept of terroir [67]. However, there is an opportunity to avoid the mistakes of the *terroir* system—the false importance of the elite landowners [68], the neglect of worker contribution [69] and the omission of key power relationships, such as gender and kinship [70]. With government support and, crucially, the participation and drive of local villagers, the Tai Lue people can forge a place for their own foodscape in the new tourism environment. Future research and initiatives must be conducted to harness this opportunity and consider ways in which centralization, heightened awareness and increased stakeholder trust may lead to the success of regional development, as well as ways in which they may restrict opportunities, traditional culture and local ownership [51, 52].

There were a number of obstacles arising during the implementation of the research. Firstly, the project received approval in March 2020, at the height of the COVID-19 pandemic. As a result, the first phase of action research (March–April) could not be completed thoroughly. Moreover, taste-testing and data collection at the Creative Lanna Festival could not happen. Furthermore, interview appointments sometimes had to be postponed suddenly due to illness and important events in the community, such as funerals, which could not be predicted in advance. It is hoped that there may be less disruption to any future investigations. The research team has identified some unaddressed issues, which offer the opportunity for further research. This was a geographically limited exploration of the Tai Lue cuisine that could be expanded to consider other Tai Lue ethnic communities, which are widely distributed in the north. Concrete opinion from external visitors regarding the nature of the original and transformed Tai Lue cuisine is crucial for the efficiency of further development. Therefore, market research is highly recommended. Importantly, the Tai Lue communities must be supported to support themselves, especially in communication and publicity. Food may be the gateway attraction to Tai Lue culture, but there are many exciting elements of the culture for visitors to experience. This process was begun with the podcast sessions, but requires further training of local people so that they may create and implement similar projects. It is equally important to promote the transmission of traditional knowledge to youth in the area so they can be the leaders of future prosperity and drive the community to be strong and sustainable.
